# The Clinical Application of Fluorescence-Guided Surgery in Head and Neck Cancer

**DOI:** 10.2967/jnumed.118.222810

**Published:** 2019-06

**Authors:** Stan van Keulen, Naoki Nishio, Shayan Fakurnejad, Andrew Birkeland, Brock A. Martin, Guolan Lu, Quan Zhou, Stefania U. Chirita, Tymour Forouzanfar, A. Dimitrios Colevas, Nynke S. van den Berg, Eben L. Rosenthal

**Affiliations:** 1 Division of Head and Neck Surgery, Department of Otolaryngology, Stanford University School of Medicine, Stanford, California; 2 Department of Oral and Maxillofacial Surgery/Oral Pathology, VU University Medical Center/Academic Centre for Dentistry Amsterdam (ACTA), Amsterdam, The Netherlands; 3 Department of Clinical Pathology, Stanford University School of Medicine, Stanford, California; 4 Cancer Clinical Trials Office, Stanford Cancer Center, Stanford University School of Medicine, Stanford, California; and; 5 Division of Medical Oncology, Department of Medicine, Stanford University School of Medicine, Stanford, California

**Keywords:** fluorescence-guided surgery, head and neck cancer, real-time intraoperative imaging

## Abstract

Although surgical resection has been the primary treatment modality of solid tumors for decades, surgeons still rely on visual cues and palpation to delineate healthy from cancerous tissue. This may contribute to the high rate (up to 30%) of positive margins in head and neck cancer resections. Margin status in these patients is the most important prognostic factor for overall survival. In addition, second primary lesions may be present at the time of surgery. Although often unnoticed by the medical team, these lesions can have significant survival ramifications. We hypothesize that real-time fluorescence imaging can enhance intraoperative decision making by aiding the surgeon in detecting close or positive margins and visualizing unanticipated regions of primary disease. The purpose of this study was to assess the clinical utility of real-time fluorescence imaging for intraoperative decision making. **Methods:** Head and neck cancer patients (*n* = 14) scheduled for curative resection were enrolled in a clinical trial evaluating panitumumab-IRDye800CW for surgical guidance (NCT02415881). Open-field fluorescence imaging was performed throughout the surgical procedure. The fluorescence signal was quantified as signal-to-background ratios to characterize the fluorescence contrast of regions of interest relative to background. **Results:** Fluorescence imaging was able to improve surgical decision making in 3 cases (21.4%): identification of a close margin (*n* = 1) and unanticipated regions of primary disease (*n* = 2). **Conclusion:** This study demonstrates the clinical applications of fluorescence imaging on intraoperative decision making. This information is required for designing phase III clinical trials using this technique. Furthermore, this study is the first to demonstrate this application for intraoperative decision making during resection of primary tumors.

See an invited perspective on this article on page 756.

Surgical resection is one of the cornerstones of therapy for patients with head and neck squamous cell carcinomas (HNSCC). Moreover, the most important factor for predicting long-term cancer survival is the completeness of the surgical resection (1–4). Despite this awareness, between 15% and 30% of oral cavity cancer patients have positive surgical resection margins after surgery, which is associated with poor outcomes and necessitates additional therapy (1,5,6). Furthermore, there can be concomitant primary malignancies that are often undetected at the time of the surgical resection. Notably, additional primary malignancies represent the second leading cause of death in patients with HNSCC (7).

For centuries, surgeons have relied exclusively on visual and tactile cues during surgical resection. However, tumors, and in particular tumor margins, remain challenging to ascertain. The subjective nature of the resection can be especially challenging in the oral cavity, due to a small working area and proximity of critical structures that are at risk for injury. The current strategies of detecting tumor margins during resection have demonstrated that the surgeon has only a 36% accuracy to detect true-positive margins (8). Recognizing this, several attempts have been made to develop techniques for assessment of tumor tissue during the surgery that does not solely rely on visual and tactile cues. The current standard for detecting residual disease is gross inspection of the surgical specimen or wound bed, followed by frozen sectioning analysis of suspicious areas (9). Besides the time-consuming nature of the procedure (15–20 min per frozen section), frozen section analysis can only examine a small fraction of the specimen (9). Consequently, alternative real-time intraoperative imaging techniques have been proposed to assist the surgeon in decision making, including ultrasound, radiofrequency spectroscopy, Raman spectroscopy, optical coherence tomography, and photoacoustic imaging (10–12).

Recently, there has been a rapid growth in development of optical contrast agents for the real-time assessment of tumors during surgery using fluorescently labeled, tumor-specific probes (13–16). In the current study, we ask if intraoperative visualization of tumor margins and occult cancer can be performed using fluorescently labeled antibodies to improve the rate of successful resection. Despite the large number of clinical trials that have identified the safety and feasibility of tumor-targeting optical imaging agents, only a limited number of publications have successfully demonstrated their clinical value (17–19). The objective of this study was to assess the clinical value of real-time fluorescence imaging during surgery to guide intraoperative decision making.

## MATERIALS AND METHODS

### Study Design

Fourteen patients with biopsy-proven HNSCC scheduled to undergo surgical resection with curative intent were included in our ongoing phase I study assessing panitumumab-IRDye800CW. These patients received an intravenous infusion of panitumumab-IRDye800CW 1–5 d before surgery as previously described (8). Panitumumab-IRDye800CW is a near-infrared fluorescence imaging agent with an excitation/emission maximum at 774/789 nm and a half-life of approximately 24 h (13) and a maximal observed penetration depth of 6.3 mm (20). At the time of surgery, intraoperative fluorescence imaging was performed at 4 stages during the surgery using a dedicated hand-held near-infrared fluorescence imaging device (Novadaq) specialized for the detection of IRDye800. Throughout the surgery, image acquisition was performed intermittently at different stages during the procedure. First, the surgical field was imaged before incision to demarcate the primary tumor and screen for potential other primary lesions. Next, during the resection the surgical field was imaged to visualize the deep surgical margin (the cut surface on the primary specimen). After primary tumor resection, the wound cavity was imaged to potentially visualize any residual disease. Last, the entire surface of the surgical specimen was imaged ex vivo to assess the surgical margins on the tumor specimen. Throughout image acquisition, camera settings were kept consistent and the overhead lights were turned off. The study protocol was approved by the Stanford University Institutional Review Board (IRB 35064) and the Food and Drug Administration (NCT02415881), and written informed consent was obtained from all patients. The study was performed in accordance with the Helsinki Declaration of 1975 and its amendments, Food and Drug Administration’s International Conference on Harmonisation–Good Clinical Practice guidelines, and the laws and regulations of the United States.

### Fluorescence Analysis

To estimate signal-to-background ratios (SBRs) in the image presented to the surgeon, images were loaded into ImageJ (version 1.50i; National Institutes of Health) where regions of interest were drawn around tissue areas of interest. In line with previously published literature (8,21–23), the estimated SBR was calculated by dividing the mean signal intensity (MSI) of the region of interest drawn around the area of interest (i.e., tumor or wound bed) by the MSI of the background signal (i.e., nearby normal tissue).

A background value was estimated from 10 regions of interest for different tissue types (i.e., tongue, gingival and buccal mucosa) in the oral cavity for each patient, with each region of interest located at least 3–4 cm from the edge of the gross tumor. An average background was identified by comparing the MSI and variance in MSI for all tissue types (tongue, gingival and buccal mucosal tissue) before and after resection of the primary tumor specimen. The variance in signal was defined as the coefficient of variance (CV), which is the SE divided by MSI and describes the heterogeneity of the tissue (e.g., tumor often has high variation in signal and thus a high CV). Subsequently, the tissue type with the most constant signal and CV was selected as background.

### Histologic Assessment

Intraoperative fluorescence-guided tissue sampling through frozen sectioning was performed per standard of care. Final histopathologic assessment of the tissue specimens was conducted by a board-certified pathologist after routine hematoxylin and eosin (H&E) staining. To assess the distance from the tumor border to the cut edge of the specimen on the deep aspect of the specimen, known as the deep margin, the pathologist outlined regions of tumor on the H&E slides. Thereafter, the H&E slides were imaged using an Odyssey imaging platform (LI-COR Biosciences) to identify fluorescence signal within the tissue, which was later correlated with in vivo imaging.

## RESULTS

### Variation in Fluorescence per Tissue Type

Of the 14 patients with HNSCC who were included in this study, a total of 700 data points were obtained from the acquired intraoperative fluorescence images. For background fluorescence level establishment, we found that besides being visually different, each background tissue type, including normal tongue and gingival and buccal mucosal tissue, had its own MSI range and distribution pattern of signal (CV). The tissue’s unique MSI and CV allowed surgeons in the study to discriminate the different tissue types (Supplemental Fig. 1; supplemental materials are available at http://jnm.snmjournals.org). Buccal mucosal tissue was selected as the optimal background because it showed the least change in MSI and subsequent CV. The visual fluorescence signal was also most homogeneous when compared with normal tongue and gingival tissue. With buccal mucosal tissue serving as the background, the SBRs of the primary tumors were found to be much higher than those of the wound cavities (SBRs ranging from 1.8 to 2.7 for tumors versus 0.2 to 0.7 for wound cavities).

### Clinical Value of Fluorescence Imaging During Surgery

Of all studied cases, we found that fluorescence imaging improved surgical decision making in 3 cases (21.4%). Improved surgical decision making is defined as instances when the fluorescence imaging information changes the surgical procedure to ensure better surgical outcome. Table 1 summarizes the clinical value of fluorescence imaging during the surgical procedure for each patient. In all cases, real-time fluorescence imaging of the tumor before surgery successfully outlined the tumor as defined by histology. Furthermore, in some cases, visualization of unrecognized tumor led to modification of the planned borders of the surgical resection. Specific use of fluorescence imaging is further discussed in the following paragraphs.

**TABLE 1:** Patient and Tumor Characteristics

			Fluorescence assessment and potential benefit					
			Tumor			Margins	
Patient no.	Tumor site	Tumor stage	Fluorescent visualization*	SBR^†^	Detection of secondary lesion^‡^	Successful presentation of peripheral margin^¶^	Successful presentation of deep margin^§^	Detection of residual disease^‖^
1	Lateral tongue	pT2N0M0	Yes	1.92	—	+	+	—
2	Lateral tongue	pT3N2cM0	Yes	2.03	—	+	+	—
3	Retromolar trigone	pT3N0M0	Yes	2.38	—	—	—	—
4	Buccal mucosa	pT2N2bM0	Yes	2.68	+	+	+	—
5	Buccal mucosa	pT3N0M0	Yes	2.55	—	+	+	—
6	Hard palate	pT2N0M0	Yes	2.03	—	+	—	—
7	Lateral tongue	pT2N2bM0	Yes	1.77	—	+	+	—
8	Floor of mouth	pT3N2bM0	Yes	1.50	—	+	+	—
9	Retromolar trigone	pT4aN2bM0	Yes	1.56	—	—	—	—
10	Lateral tongue	pT2N0M0	Yes	2.34	—	+	+	—
11	Lateral tongue	pT1N0M0	NA	NA	—	+	+	—
12	Maxillary sinus	pT4N0M0	Yes	2.30	—	—	—	—
13	Scalp	NA	NA	NA	—	—	+	—
14	Primary unknown	pTxN3bM0	NA	NA	—	+	—	+

* Fluorescent visualization of primary tumor. Patients 11, 13, and 14 had no primary tumor.

† SBRs in patients 11, 13, and 14 could not be calculated in absence of primary tumor.

‡ Discovery of novel secondary primary tumors in the oral cavity.

¶ Fluorescent assessment of mucosal surface to screen close/positive margin (<5 mm).

§ Fluorescent assessment of deep surface to screen close/positive margin (<5 mm).

‖ Detection of residual disease to biopsy and correlate with pathologic findings.

### Real-Time Deep Margin Assessment

**FIGURE 1.**
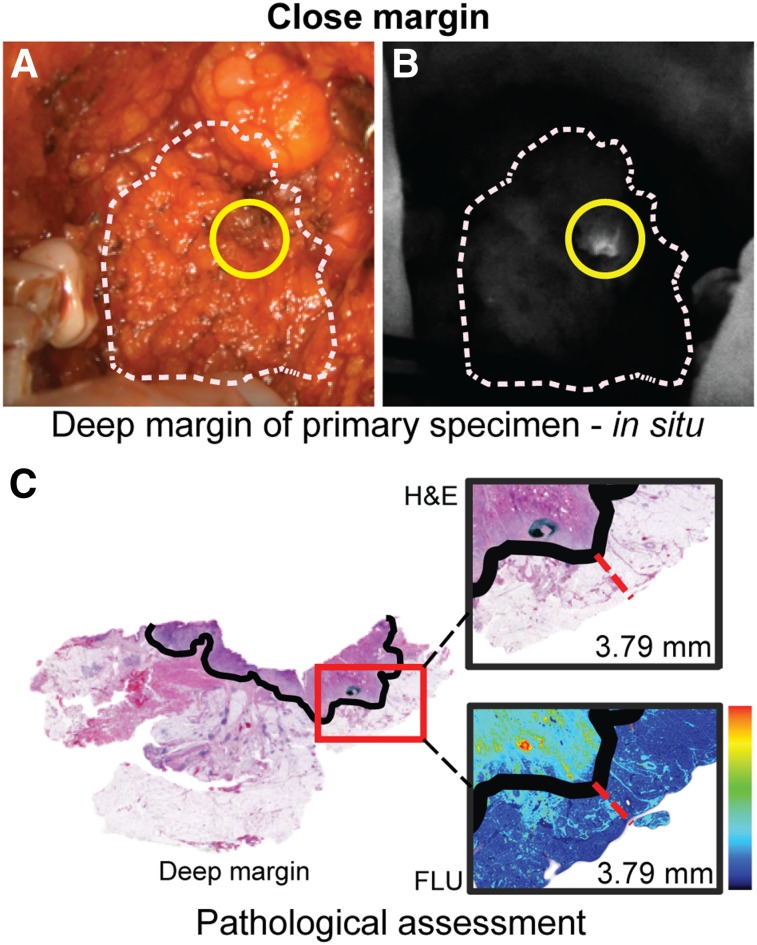
Fluorescence-guided deep margin assessment. This figure illustrates a case in which a close deep margin was detected using fluorescence imaging. (A and B) In situ bright-field (A) with corresponding fluorescence image (B). Yellow circle marks close deep margin. (C) Measured distance of tumor border (black solid line) to deep margin on H&E slide with zoomed-in bright-field image and corresponding fluorescence image. FLU = fluorescence image.

Although remaining a topic of debate in head and neck surgery, a margin is often considered positive if there is tumor present within 2 mm of the edge of the surgical specimen, close if there is tumor present within 2–5 mm, and negative if tumor is further than 5 mm from the surgical specimens’ edge (4). Gross assessment of the deep margin (defined as the distance from the tumor border to the cut edge of the specimen on the deep aspect of the specimen) remains challenging due to variations in tumor depth and subtle tissue changes associated with tumor extension. We were able to accurately assess the deep margin using fluorescence imaging in 10 patients with tumor invading soft structures (71.4%). Assessment of the deep margin in patients with cancer adherent to bone (retromolar trigone squamous cell carcinoma [SCC] [*n* = 2], maxillary sinus SCC [*n* = 1], or palate SCC [*n* = 1]) remained difficult, partly because the open-field devices are not currently designed for deep wound cavity imaging. In 9 of 10 patients, the imaged deep margin of the tumor was negative for fluorescence, and the tumor margins were later confirmed to be greater than 5 mm on final histopathology (average distance, 7.6 mm; range, 5–15 mm; Supplemental Fig.2). The remaining patient presented with a buccal lesion that revealed a region of high fluorescence signal when viewed from the deep margin during resection (Fig. 1). After histologic evaluation of the H&E slide, this fluorescence-positive deep margin was found to contain tumor within 3.8 mm from the surgical specimens’ edge (Fig. 1C).

### Visualization of Unanticipated Regions of Primary Disease

**FIGURE 2.**
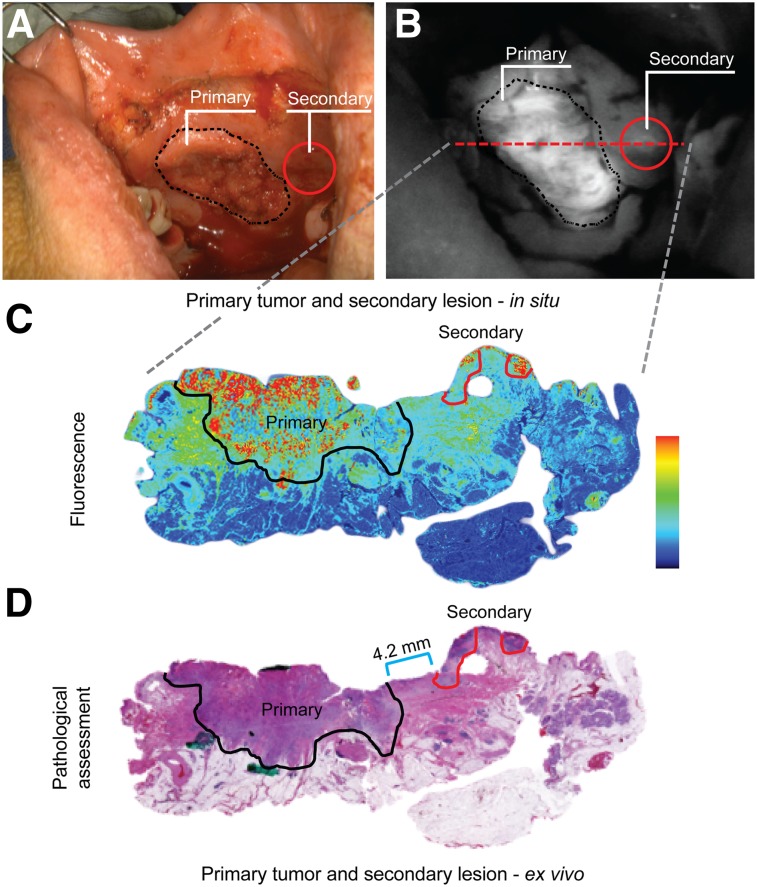
Detection of secondary primary. (A and B) In situ bright-field (A) and corresponding fluorescence image (B) of primary tumor (black dotted line) and secondary tumor (red circle). Red dashed line indicates location from which H&E slide was obtained. (C and D) Shown are fluorescence image (C) and corresponding H&E slide image (D) with measured distance (blue bar) from primary tumor (black solid line) to secondary tumor (red solid line). Primary = primary tumor; secondary = secondary tumor.

Second primary lesions are common in HNSCC and often go unnoticed by the surgical team. In 1 case, fluorescence imaging of buccal SCC, before the surgical incision, led to identification of such a secondary lesion outside the planned surgical incision (Fig. 2). On the basis of this intraoperative finding, the surgeon extended the surgical incision to include the suspicious lesion that correlated with the location of the fluorescence signal. Quantitative assessment of the lesion indicated an SBR greater than 2, both for in situ and ex vivo imaging. Final pathologic evaluation of the second lesion revealed an invasive SCC that was separated from the primary tumor by a bridge of 4.2-mm normal mucosa.

**FIGURE 3.**
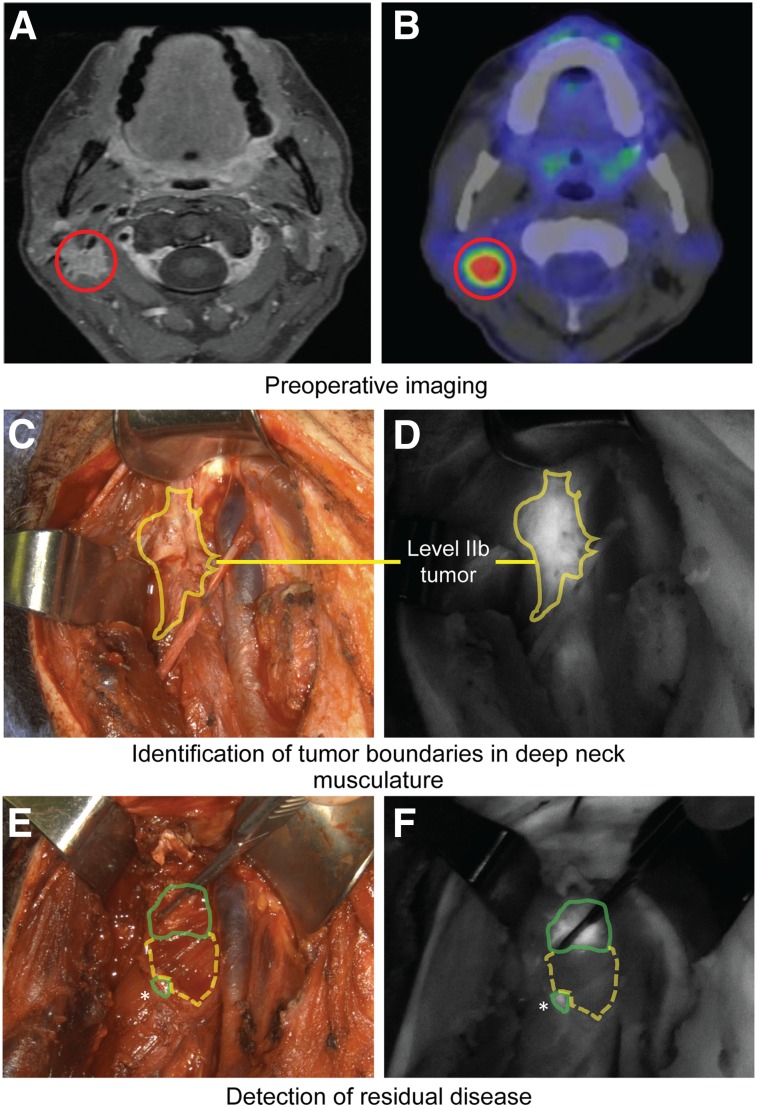
Detection of unanticipated regions of primary disease. (A and B) Shown are MRI (A) and ^18^F-FDG PET (B) images of level II lesion (red circles). (C and D) After removal of level IIa LNs, extent of level IIb tumor (yellow solid line) became visible using fluorescence imaging. (E and F) Detection of residual disease (green solid line) surrounding removed tumor mass (yellow dashed line). *Residual tumor tissue that had otherwise gone unnoticed.

Regional metastasis with extracapsular extension often requires complex surgical intervention. In 1 case, preoperative MRI revealed a suspicious lymph node (LN) and an indistinct mass in level II of the right neck, as well as a suspicious LN in level V of the right neck. Although not uncommon (24), PET imaging only disclosed a solitary ^18^F-FDG–avid spot in neck level II (Fig. 3) that was positive on fine-needle aspiration. Intraoperative fluorescence imaging demonstrated several fluorescent LNs in level II as well as the level V LN that was seen on preoperative MRI. Repeated fluorescence imaging was particularly valuable for the visualization of the extent of the level II mass, which was found to have infiltrated the deep neck musculature. On complete gross resection of this mass, it was found that fluorescence imaging allowed for the identification of multiple small pieces of residual tissue that were not detected by the surgeon’s gross inspection (SBRs > 2; Fig. 3). Pathologic assessment of these tissue samples by frozen section analysis confirmed SCC.

### Assessment of Wound Cavity

After complete gross surgical resection of the primary tumors, fluorescence imaging of the wound was performed. In all wound cavities, the estimated SBR remained below 1 (ranging from 0.2 to 0.7), indicating that the signal in the wound cavity was never higher than that of the background signal (i.e., buccal mucosal tissue). Final histopathologic assessment of the resected specimens revealed no positive margins, indicating that no tumor tissue was left in situ.

## DISCUSSION

Surgeons traditionally rely on visual inspection of subtle surface changes and palpation to determine tumor margins. Findings from our current study suggest that open-field fluorescence imaging can improve detection of tumor and tumor margins. Our data suggest that fluorescent imaging can be used to evaluate the primary tumor, surrounding mucosa, and regional metastatic disease during ablative resection. We believe that our findings illustrate scenarios in which surgical experience, visualization, and palpation can be successfully augmented with fluorescence imaging to improve clinical care and patient outcomes.

Quantifying imaging data remained challenging because open-field devices are not used in a light-controlled environment where ambient light, distance, and signal can be standardized. Furthermore, the surgeon uses the real-time information throughout the case, continuously incorporating the fluorescence data with tactile information, white light images, and experience. As a result, isolating the value of the imaging information can be difficult to assess objectively.

We have sought to identify 2 different strategies to assess the value of real-time imaging; one in which disease can be visualized encroaching on the deep margin of the tumor and the other in which disease is outside expected boundaries. These findings are uniquely valuable in that imaging information leads to immediate reevaluation of the surgical site, preventing a close or microscopically positive margin. Our findings are consistent with previously published results. The randomized-controlled study by Stummer et al. (18) reported that fluorescence visualization of malignant glioma during surgery resulted in a significant increase in complete resection (65% vs. 36%, *P* < 0.0001) and subsequently fewer reinterventions. Clinical trials such as these will be critical to show the value of these real-time open-field techniques.

Previously, we demonstrated the safety, sensitivity, and specificity of antibody-fluorescence dye for surgical imaging (8,13). Also, we demonstrated that closed-field ex vivo imaging of the surgical specimen has the advantage over open-field in situ imaging due to less reflectance and no interference of ambient light (8). Closed-field systems can be used for optical mapping of the surgical specimen in a highly sensitive and quantitative fashion to identify suspicious areas that may guide pathologic assessment. Nevertheless, closed-field systems are incapable of in situ disease assessment. Therefore, open-field systems are needed for in situ evaluation of disease extent and assessment of close and positive deep margins in real time.

Although open-field imaging technologies have advanced significantly, important limitations must be considered. Although this study demonstrates the potential utility of real-time fluorescence imaging for surgical tumor resection, the true value of this technique will be seen when patient outcome data become available. Other limitations encountered during this study offer important insight in the value of open-field devices for surgical navigation. In their current form, imaging results are not quantitative using open-field devices because the instruments are influenced by ambient light in the operating room environment, camera angle, and distance between the camera and the patient. To obtain quantifiable imaging information, a controlled environment using a closed-field fluorescence imaging device is needed, which requires an ex vivo setting (20). Currently, some open-field systems are able to suppress a significant amount of ambient light by synchronizing the acquisition to the 120 Hz of room light with pulsed LED excitation (25). Furthermore, to be widely applicable, software adaptations have to allow the camera to accommodate in a wide range of signal intensities and distances. Although this will enable small fragments of tumor to be distinguished from the background, various contrast-enhancement schemes may also increase the estimated SBR for nonspecific structures in the absence of a definitive high-intensity signal (such as tumor). We also believe that in order for open-field systems to be successful, the surgeon’s experience and other operative information must be integrated with use of the camera system. Tumor signals appear highly heterogeneous, compared with the uniform, smooth appearance of the mucosal signal. We showed that different tissue types have unique fluorescent patterns (visually, MSI and CV), which can be incorporated into the surgeon’s armamentarium to distinguish normal from cancerous tissue. Routine use of fluorescence imaging may permit development of pattern-recognition skills to identify suspicious areas or to distinguish tumor from off-target signal in a fashion similar to the pattern-recognition skills that radiologists use when interpreting anatomic imaging. Consistent with this analogy, radiologists often identify specific tissues based on their radiographic appearance (Supplemental Fig. 1, similar to salt-and-pepper signals in MRI literature (26)). We predict that as fluorescence imaging further develops into the clinic, software and hardware improvements, pattern recognition, and background identification could be used to set a baseline for imaging at the beginning of the case. In this manner, a patient-specific, fixed threshold could be established and used to quantify suspicious areas throughout the whole case. Furthermore, future studies might involve the use of machine-learning approaches to delineate tumor from healthy tissue based on signal heterogeneity and SBR.

## CONCLUSION

In this study, we demonstrated potential utilities of real-time fluorescence imaging for intraoperative guidance in oncologic head and neck surgery. Furthermore, we proposed modifications for future open-field camera systems to augment successful surgical resection and improvement of patient outcome.

## DISCLOSURE

This work was supported in part by the Stanford Comprehensive Cancer Center, the Stanford University School of Medicine Medical Scholars Program, the Netherlands Organization for Scientific Research (Rubicon; 019.171LW.022), the National Institutes of Health and the National Cancer Institute (R01CA190306), the Stanford Molecular Imaging Scholars (SMIS) program (T32 CA118681), and an institutional equipment loan from Novadaq. Eben Rosenthal is a consultant for Novadaq and has equipment loans from this company. No other potential conflict of interest relevant to this article was reported.
