# Supplementary material for: The Clinical Application of Fluorescence-Guided Surgery in Head and Neck Cancer
Source: J Nucl Med. 2019 Jun;60(6):758–63. doi: 10.2967/jnumed.118.222810 (PMC6581234; doi:10.2967/jnumed.118.222810)
Supplement: Supplementary file 1 [file jnm222810SupplementalData.pdf]

SUPPLEMENTAL FIGURE 1

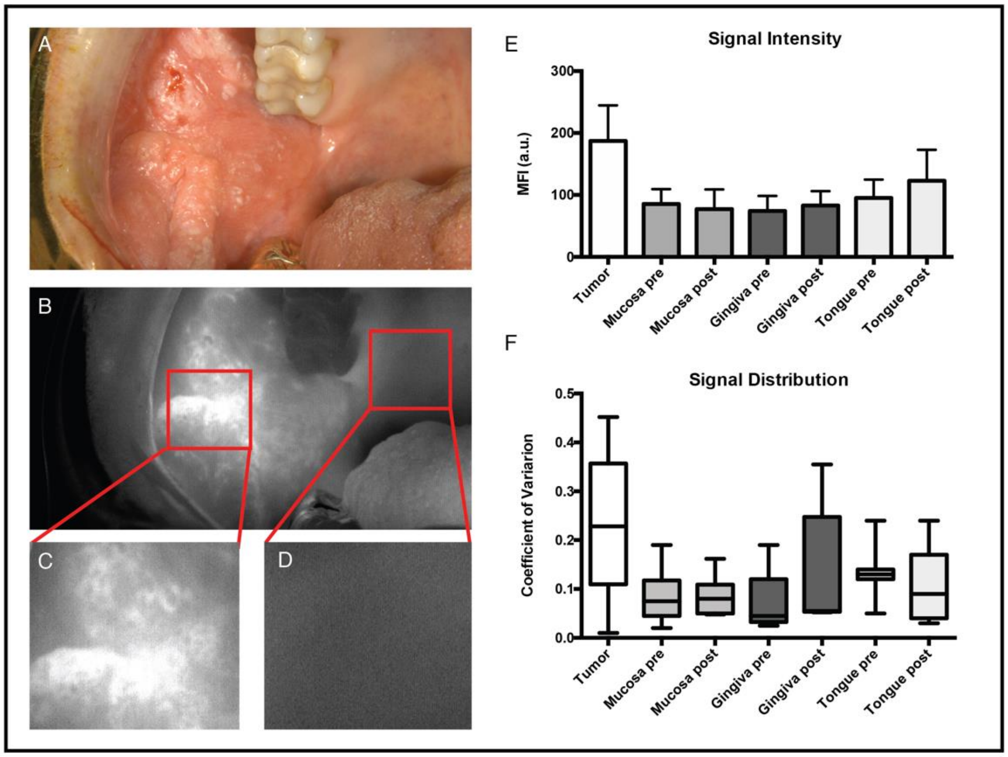

SUPPLEMENTAL FIGURE 1 Fluorescence tissue patterns. (A and B) Bright field (A) and corresponding fluorescence image (B) of a representable patient with a buccal mucosa squamous cell carcinoma. (C and D) Close up of heterogenetic tumor tissue (C) and mucosal tissue (D). (E and F) Represents the signal intensity (E) and signal distribution (F) for each tissue type. a.u. = arbitrary units; MSI = mean signal intensity; pre = pre-resection; post = post resection.

SUPPLEMENTAL FIGURE 2

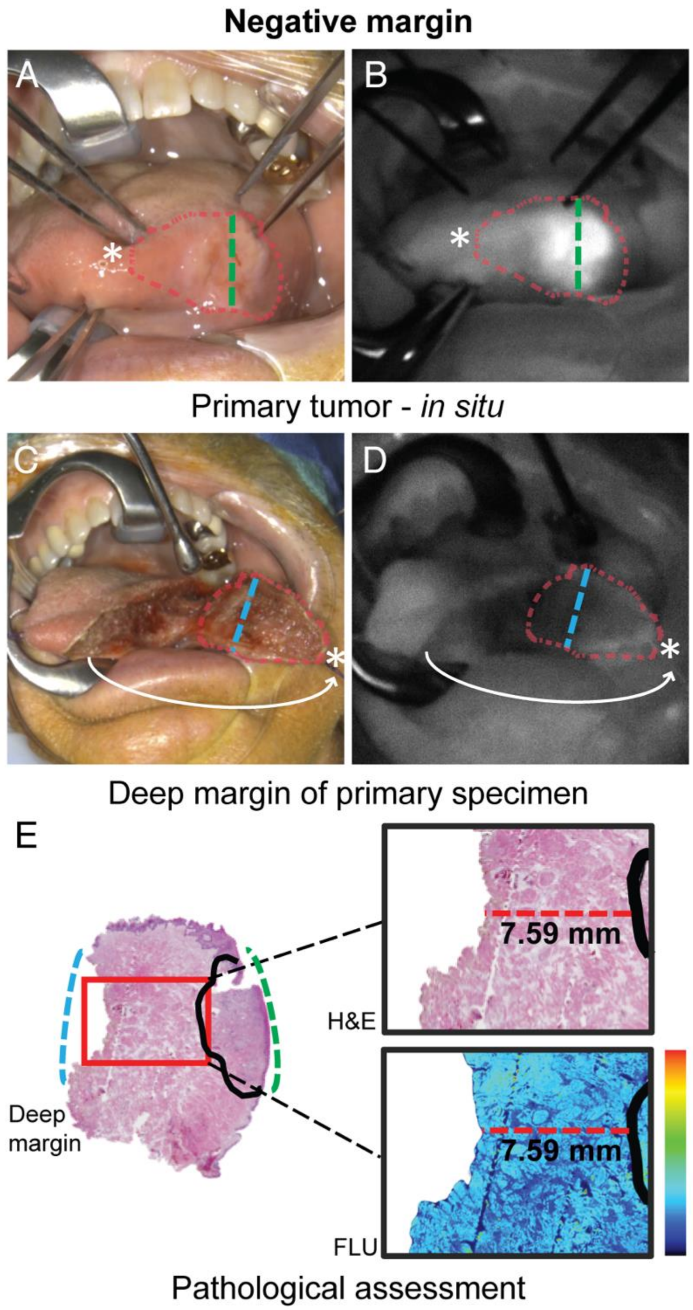

SUPPLEMENTAL FIGURE 2 Fluorescence-guided deep margin assessment. Figure shows negative deep margin. (A and B) Planned surgical cut (red dotted line) and mucosal surface (green dotted line) in bright-field (A) with corresponding fluorescence image (B). (C and D) After incision the

deep margin (blue dotted line) is opened up with forceps (asterisk with arrow). C shows the bright-field image and D the corresponding fluorescence image. (E) Measured distance of tumor border (black solid line) to deep margin on the hematoxylin and eosin (H&E) slide. The green and blue dotted lines represent the mucosal margin and deep margin, respectively, with zoomed-in bright-field H&E image and corresponding fluorescence image.
